# A Discovery-Based Metabolomic Approach Using UPLC-Q-TOF-MS/MS Reveals Potential Antimalarial Compounds Present in *Artemisia annua* L.

**DOI:** 10.3390/ijms232314903

**Published:** 2022-11-28

**Authors:** Henan Shi, Zhuqing Wang, Fujie Xu, Jialin Li, Jing Li, Manyuan Wang

**Affiliations:** Beijing Key Laboratory of TCM Collateral Disease Theory Research, School of Traditional Chinese Medicine, Capital Medical University, Beijing 100069, China

**Keywords:** *Artemisia annua* L., synergistic effect, antimalarial, UPLC-Q-TOF-MS, OPLS-DA

## Abstract

In 1972, Nobel laureate Youyou Tu’s research team conducted clinical trials on the dried material of *Artemisia annua* L. from Beijing extracted by ether and then treated with alkali (called “ether neutral dry”), which showed that artemisinin was not the only antimalarial component contained. The biosynthesis of sesquiterpenoids in *A. annua* has increased exponentially after unremitting cultivation efforts, and the plant resources are now quite different from those in the 1970s. In consideration of emerging artemisinin resistance, it is of great theoretical and practical value to further study the antimalarial activity of *A. annua* and explore its causes. The purpose of this study is to clarify scientific questions, such as “What ingredients are synergistic with artemisinin in *A. annua?*”, and “Are there non-artemisinin antimalarial ingredients in *A. annua?*”. In this paper, Beijing wild *A. annua* was used as a control and two representative cultivation species of *A. annua* were selected to evaluate the antimalarial activity of the herbal medicine. The antimalarial activity of different extracts on mice was studied using the Peters’ four-day suppressive test. UPLC-Q-TOF-MS was used to obtain mass spectrum data for all samples, and a UNIFI platform was used for identification. A multivariate statistical method was used to screen the different compounds with positive correlations. The antimalarial activity of different components from the ether extract and alkali treatments was determined and antimalarial components other than artemisinin were obtained. A total of 24 flavonoids, 68 sesquiterpenoids and 21 other compounds were identified. Compounds associated with differential antimalarial activity were identified. The material basis for the antimalarial activity of *A. annua* was clarified. The antimalarial components of *A. annua* include two categories: first, artemisinin and non-artemisinin antimalarial active components, of which the non-artemisinin antimalarial active components may include 5α-hydroperoxy-eudesma-4(15),11-diene; second, several antimalarial synergistic ingredients in *A. annua*, including arteanniun B, arteanniun B analogues and polymethoxy flavonoids.

## 1. Introduction

According to the World Malaria Report 2021, there were about 241 million malaria cases in 85 malaria-endemic countries (including French Guiana) in 2020, an increase of 44% compared to the previous year. The number of deaths caused by malaria is on the rise. Artemisinin-based combination therapies (ACTs) have been widely used since 2001 [1]. By 2006, resistance had developed to all classes of antimalarial drugs except artemisinin derivatives. The first edition of malaria treatment guidelines released by WHO strongly advocated the use of artemisinin combination therapy, specifically recommending four combinations, which have gradually become first-line treatment drugs [2]. The second edition of the malaria treatment guidelines published in 2010 added the combination of dihydroartemisinin + piperaquine as the fifth most recommended combination [3]. However, in recent years, the research and development of new malaria drugs with drug resistance benefits has made only slow and non-ideal progress. In the third edition of the malaria treatment guidelines released in 2015, the main drug combinations were still five in number, and there were no new drug combinations for malaria prevention and control [4]. From 2015 to 2017, signs of artemisinin resistance of *Plasmodium falciparum* were successively confirmed in the Great Mekong Sub-region, and high numbers of cases of treatment failure were often reported after ACTs treatment [5,6]. The 2021 World Malaria Report noted that some African regions also showed signs of artemisinin resistance [7]. ACTs have remained the best treatment for malaria to date. Faced with the risk of *Plasmodium* developing resistance to artemisinin-based drugs, many experts were concerned that once *Plasmodium* develops resistance to current ACTs on a wide scale, malaria will be left without an ideal cure.

Artemisinin is an antimalarial active ingredient discovered in *A. annua* by Youyou Tu’s research team in 1972 [8]. Artemisinin is the core compound of the best antimalarial drugs available. Artemisinin has the disadvantages of poor solubility, low bioavailability, and a high recrudescense rate of *Plasmodium*. It is generally available for clinical application in the form of artemisinin derivatives (dihydroartemisinin, artesunate, artemether, etc.), which have cured numerous malaria patients worldwide [9]. The traditional use of *A. annua* suggests that its antimalarial activity is the result of a combination of components. The in vitro antimalarial activity of compounds such as artemisinin B and artemisinic acid has been reported, but no relevant in vivo antimalarial activity has been verified [10]. So far, artemisinin has been the only clearly identified antimalarial active ingredient in *A. annua*. However, we noticed a clue in the ether neutral dry of Beijing *A. annua*. Youyou Tu’s research team applied capsules of the neutral part, ether neutral dry (i.e., ether extraction, neutral part, low temperature drying), to 30 clinical patients with malaria. The results showed that ether neutral dry could cause *Plasmodium* parasites to turn negative, with remarkable effects. Experimental studies on murine and monkey malaria models showed that the neutral part of *A. annua* ether extract treated with alkali solution had strong antimalarial efficacy. After artemisinin was removed from the ether-neutral-dry part of *A. annua* by normal silica gel separation, the black oil obtained by elution of 15% ethyl acetate (EA) in petroleum ether (PE) also showed antimalarial activity [9,11]. After more than 40 years of progress in *A. annua* cultivation, the artemisinin contained in *A. annua* resources has doubled, and the content of artemisinin in high-quality resources has reached approximately 2%. The composition of *A. annua* has changed and its ether extract and ether neutral dry may now have different antimalarial effects.

In light of the above, this study seeks to resolve scientific questions, including “What antimalarial active ingredients synergistic with artemisinin occur in *A. annua?*”, and “Are there non-artemisinin antimalarial active ingredients in *A. annua*”. First, a Beijing wild *A. annua* resource prepared with ether neutral dry was selected as a control and two representative *A. annua* species with a higher content of artemisinin were selected for the objects of study. Then, from the perspective of multi-component interaction, UPLC-Q-TOF-MS/MS qualitative and metabonomic methods were combined to evaluate the antimalarial activity of *A. annua*.

## 2. Results

### 2.1. Antimalarial Activity of Different Extracts of Artemisia annua L.

Different *A. annua* samples were prepared and the content of artemisinin was determined (Table 1). The content of artemisinin in different ether-neutral-dry samples (2% ArtL-Et_2_O, 2% ArtM-Et_2_O and 2% ArtH-Et_2_O) was lower than that in the corresponding ether extracts.

**Figure 1:**
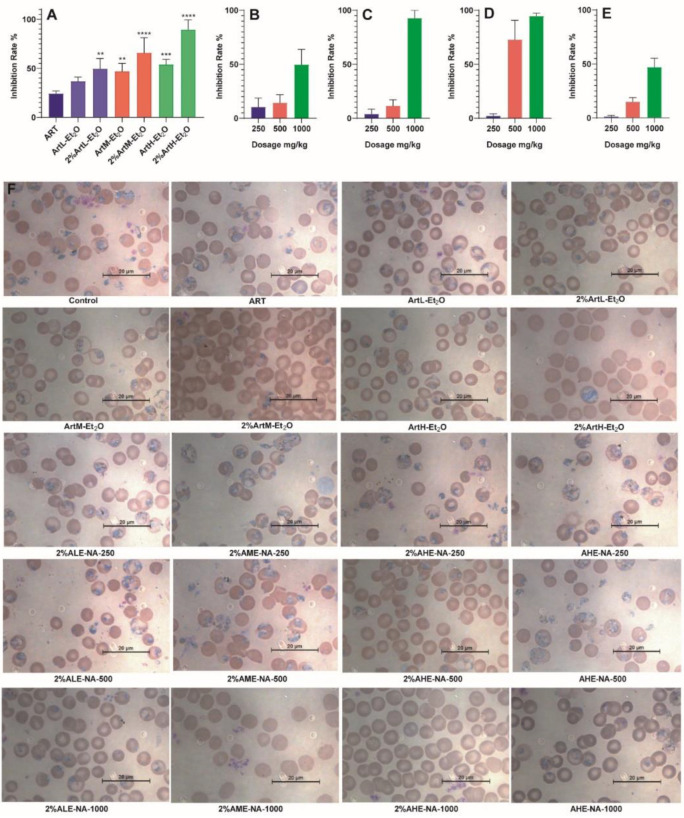
Results of antimalarial effect of different *Artemisia annua* L. parts. The data are represented as mean ± S.D., with 5 animals in each group. **, *** and **** indicate *p* < 0.01, 0.001 and 0.0001, respectively. (**A**) In vivo antimalarial efficacy of different extract groups and ether-neutral-dry groups. (**B**) In vivo antimalarial efficacy of 2% ALE-NA in different dosage groups. (**C**) In vivo antimalarial efficacy of 2% AME-NA in different dosage groups. (**D**) In vivo antimalarial efficacy of 2% AHE-NA in different dosage groups. (**E**) In vivo antimalarial efficacy of AHE-NA in different dosage groups. (**F**) Representative photomicrograph of blood smears of untreated control group and treatment groups.

As shown in Figure 1A, there was no significant difference in the antimalarial activity of ArtL-Et_2_O compared to the ART group, while ArtM-Et_2_O and ArtH-Et_2_O showed significant differences, with enhanced antimalarial activity, suggesting that the differences in the natural composition of the substances in different *A.annua* samples led to a difference in efficacy. In addition, the antimalarial activity of the ether neutral drying sample with reduced artemisinin content was also significantly higher than that of the ART group and the corresponding extracts. This suggests that the alkali treatment did not enrich artemisinin based on acidic substance removal but had other positive effects.

The 2% ALE-NA (Figure 1B), 2% AME-NA (Figure 1C) and 2% AHE-NA (Figure 1D) samples showed dose-dependent antimalarial activity, indicating the presence of non-artemisinin antimalarial components. The antimalarial effect of the 2% AHE-NA group was more obvious than that of the 2% ALE-NA and 2% AME-NA groups. In addition, AHE-NA showed antimalarial activity (Figure 1E), indicating the presence of natural non-artemisinin antimalarial active components in *A. annua*.

### 2.2. UPLC-Q-TOF-MS^E^ Qualitative Analysis

MS data was imported into the UNIFI platform for processing. According to the retention time of the reference samples and the fragmentation pathways of the different compounds in literature sources [12,13,14,15,16,17], a total of 24 flavonoids, 68 sesquiterpenoids, 17 monoterpenoids and 4 polyterpenoids were identified, as shown in Table 2 and Table 3.

### 2.3. Multivariate Statistical Analysis

#### 2.3.1. PCA

**Figure 2:**
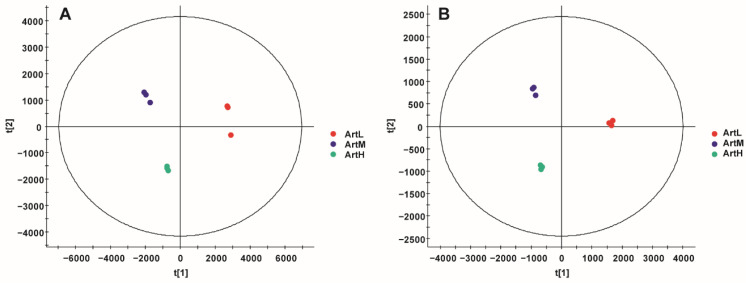
PCA score plot of the ether extracts from three *Artemisia annua* L. (**A**) positive-ion mode. (**B**) negative-ion mode (*n* = 3).

The raw positive and negative ion mass spectrometry data (raw) for the different ether extracts were imported into Progenesis QI software; effective data for 3101 and 3737 components were extracted, respectively. Non-supervisory principal component analysis was used for data difference analysis, as shown in Figure 2. The ether extract was well clustered and separated, and there were significant differences among the ether extracts. The selected samples of the different *A. annua* resources were confirmed to be representative.

#### 2.3.2. OPLS-DA

To screen out the different chemical components of *A. annua*, and further analyze the overall differences based on differences in antimalarial activity, OPLS-DA was applied to analyze ArtL-Et_2_O and ArtH-Et_2_O, 2% ArtH-Et_2_O and ArtH-Et_2_O, 2% AHE-NA and 2% ALE-NA, respectively. Based on a VIP value greater than five and an ANOVA *p* value less than 0.05, differential chemical components that were positively correlated with the efficacy results were screened out.

**Figure 3:**
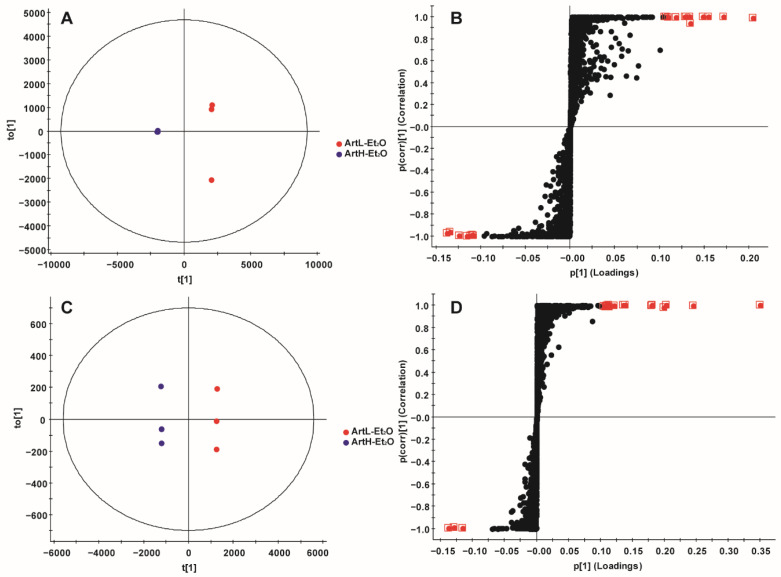
OPLS-DA of ArtL-Et_2_O and ArtH-Et_2_O. (**A**) Score plot in positive ion mode. (**B**) S-plot in positive-ion mode. (**C**) Score plot in negative-ion mode. (**D**) S-plot in negative-ion mode. Red dots represent VIP > 5 data. (*n* = 3).

Under the same absolute dosage of artemisinin, the antimalarial activity of ArtH-Et_2_O was superior to that for ArtL-Et_2_O, so components in the lower left quadrant of the S-plot should be focused on. In the positive-ion mode, artemisinin, arteanniun I, 1-oxo-2β-[3-butanone]-3α-methyl-6β-[2-propanoic acid]-cyclohexane and mikanin were the main substances associated with differences in antimalarial activity. In the negative-ion mode, arteanniun D, 4α,5α-epoxy-6α-hydroxy amorphan-12-oic acid, 1-oxo-2β-[3-butanone]-3α-methyl-6β-[2-propanoic acid]-cyclohexane were the main substances associated with differences in antimalarial activity (Figure 3, Table 4). The primary ID represents the combination of the retention time and the ion of the marker. The neutral mass and *m*/*z* in the table represent information about the markers analyzed by Progenesis QI, respectively, which represent ionic fragments of the corresponding compounds, providing a reference for the identification of the compounds.

**Figure 4:**
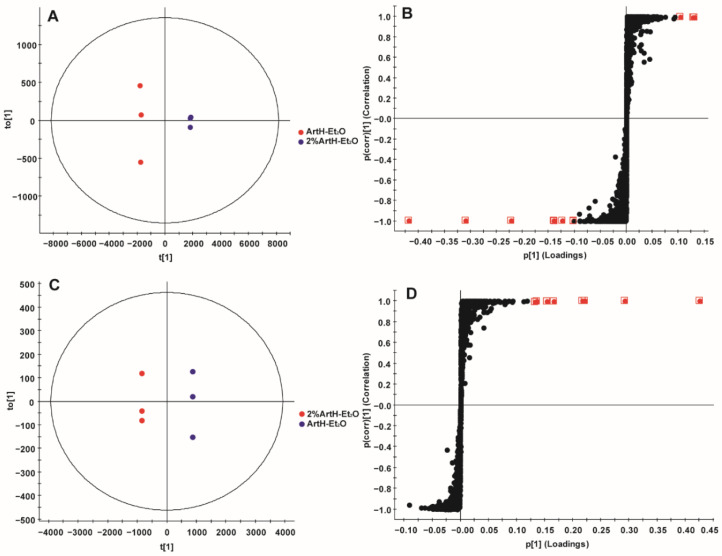
OPLS-DA of ArtH-Et_2_O and 2% ArtH-Et_2_O. (**A**) Score plot in positive-ion mode. (**B**) S-plot in positive-ion mode. (**C**) Score plot in negative-ion mode. (**D**) S-plot in negative-ion mode. Red dots represent VIP > 5 data. (*n* = 3).

Under the same absolute dosage of artemisinin, the antimalarial activity of 2% ArtH-ET_2_O was superior to that of ArtH-Et_2_O. Therefore, the components in the lower left quadrant of the S-plot should be focused on. In the positive-ion mode, arteannuin I and arteannuin K were the main substances associated with differences in antimalarial activity (Figure 4, Table 5).

**Figure 5:**
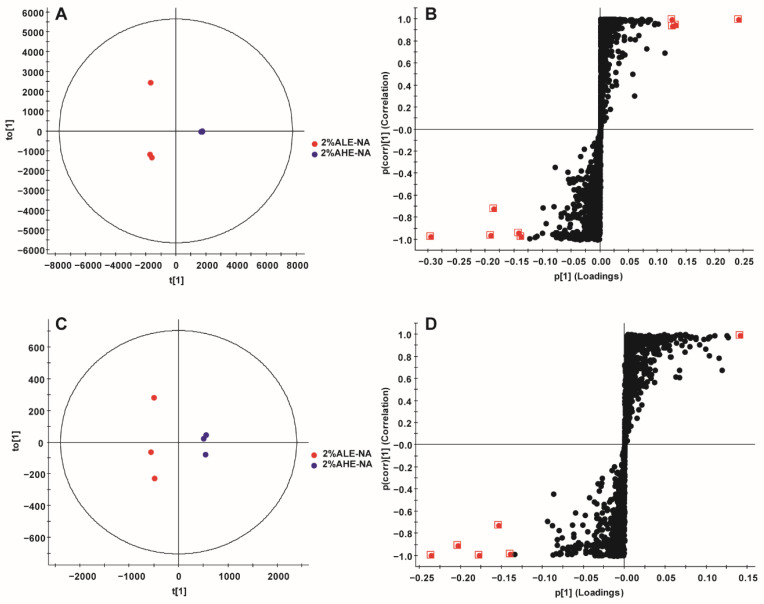
OPLS-DA of 2% ALE-NA and 2% AHE-NA. (**A**) Score plot in positive-ion mode. (**B**) S-plot in positive-ion mode. (**C**) Score plot in negative-ion mode. (**D**) S-plot in negative-ion mode. Red dots represent VIP > 5 data. (*n* = 3).

For the same dose, the antimalarial activity of 2% AHE-NA was greater than that of 2% ALE-NA, so the components in the upper right quadrant of the S-plot should be focused on. In positive-ion mode, 5-hydroxy-3,4′,6,7-tetramethoxyflavone, arteannuin L and artemetin were the main substances associated with differences in antimalarial activity (Figure 5, Table 6).

**Figure 6:**
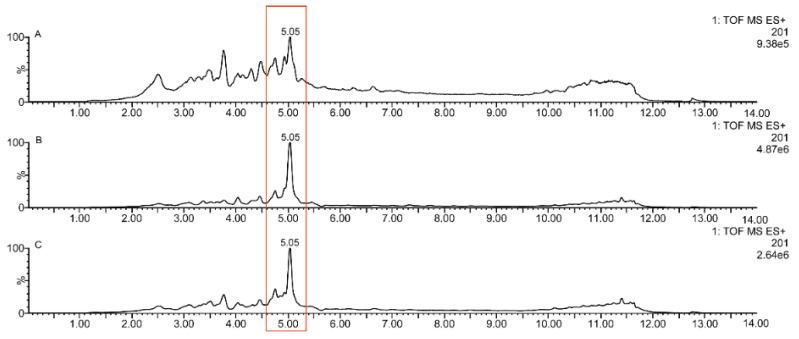
MRM chromatogram of 5α-hydroperoxy-eudesma-4(15),11-diene in three different ether-neutral-dry samples without artemisinin. (**A**) 2% ALE-NA. (**B**) 2% AME-NA. (**C**) 2% AHE-NA.

**Figure 7:**
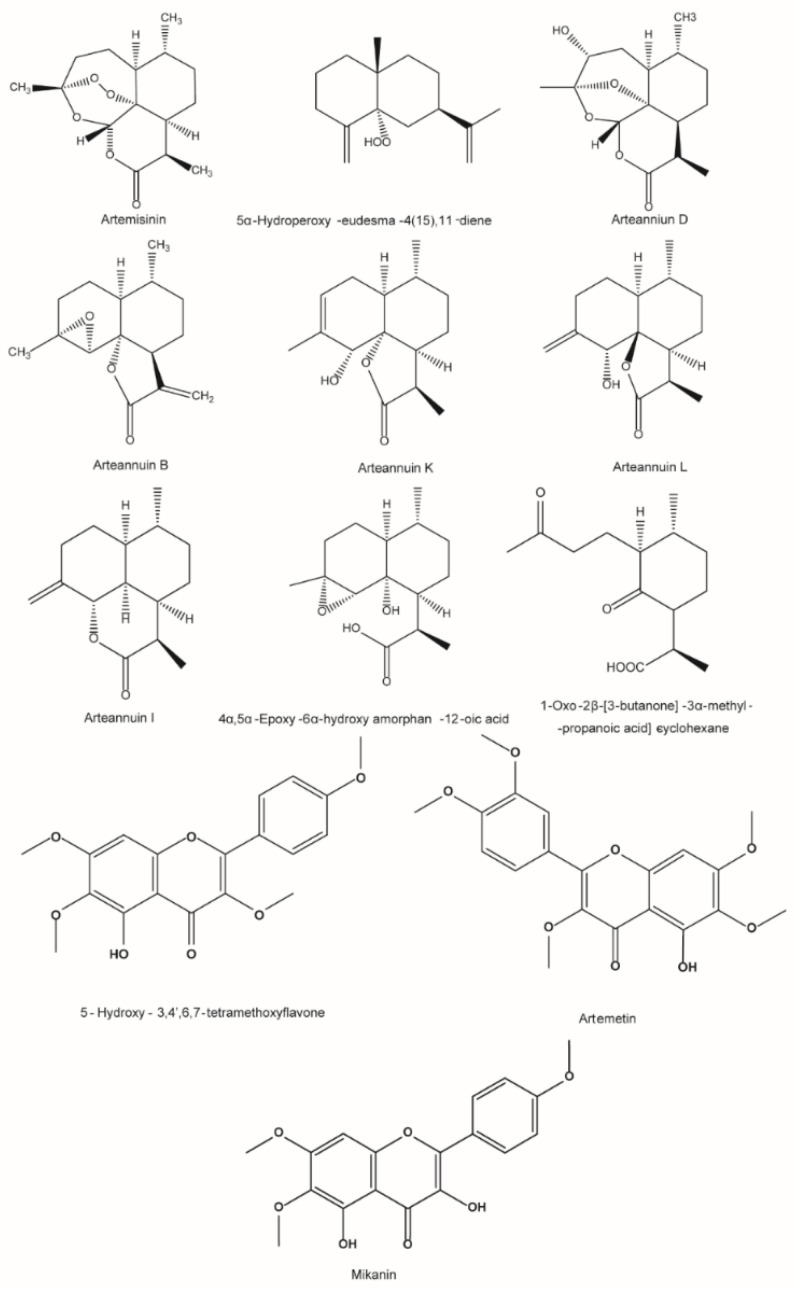
The structures of different compounds and speculated antimalarial active compounds.

The results of the in vivo efficacy tests showed that the samples without artemisinin had antimalarial activity, suggesting that other antimalarial components were present. The co-occurring compound with the strongest signal in the screened samples is shown in Figure 6, where the 5.05 min signal is presumed to be the compound 5α-hydroperoxy-eudesma-4(15),11-diene. It was observed that the antimalarial activity of artemisinin derivatives that did not have a peroxy bridge structure was also lost. The peroxy bridge is a very reactive group that can generate free radicals. Therefore, it is generally thought that the peroxy bridge structure in artemisinin plays a key role in its antimalarial activity [18]. This compound has a peroxy bond in its structure (Figure 7). It may, therefore, be one of the antimalarial components.

## 3. Materials and Methods

### 3.1. Plant Material

Beijing *A. annua*, Gansu *A. annua*, and Hunan *A. annua* were used as low artemisinin content (0.08%), denoted *A. annua* (ArtL), medium artemisinin content (1.15%), denoted *A. annua* (ArtM), and high artemisinin content (1.71%), denoted *A. annua* (ArtH), respectively. Beijing *A. annua* was self-harvested, the other samples were purchased from Hunan Siyikang Biotechnology Co., Ltd. (Hunan, China). All the above samples were identified by Professor Manyuan Wang as dry parts of the *A. annua* varieties referred to above.

### 3.2. Chemicals

Casticin and chrysosplenol D were purchased from Push (Chengdu, China) and Fifan (Shanghai, China). Arteanniun D, 1-oxo-2β-[3-butanone]-3α-methyl-6β-[2-propanoic acid]-cyclohexane methyl ester, artemisinin, arteanniun B, arteanniun C, arteanniun I, dihydroartemisinic acid, and artemisinic acid were isolated and self-made in the laboratory. The purity was above 98% (HPLC normalized purity was above 98.4%). Formic acid (LC-MS analysis) and methanol (GR) were purchased from Fisher (Waltham, MA, USA), acetonitrile (GR) from Macklin (Shanghai, China), and anhydrous ether was purchased from Beijing Chemical Works. All other reagents were analytically pure.

### 3.3. Sample Preparation

#### 3.3.1. Ether Extracts

ArtL (750 g), ArtM (500 g) and ArtH (316 g) were extracted by reflux condensation with 20 times the quantity of ether at 40 °C 3 times, for 1 h each time. After filtration, the filtrate was combined, concentrated and dried. The ether extracts ArtL-Et_2_O, ArtM-Et_2_O and ArtH-Et_2_O were obtained.

#### 3.3.2. Ether Neutral Dry

Each ether extract was extracted with 2% sodium hydroxide solution three times. The organic phase was retained, and the same volume of pure water was added to wash until neutral. Anhydrous sodium sulfate was added, and the solution stood for water absorption, filtered, concentrated and dried. Ether-neutral-dry samples of 2% ArtL-Et_2_O, 2% ArtM-Et_2_O and 2% ArtH-Et_2_O, were obtained.

#### 3.3.3. Ether Neutral Dry without Artemisinin

Each ether neutral dry sample was mixed with normal silica gel (100–200 mesh) 1:1. An amount of 10 times silica gel was packed with petroleum ether (PE, boiling range 60–90 °C, same below) into a column. Samples were then loaded using a dry method, eluted with 15% EA in PE until artemisinin and arteanniun B no longer occurred in the extracts. The remaining part of the elution by EA named ether neutral dry without artemisinin, 2% ALE-NA, 2% AME-NA and 2% AHE-NA.

In addition, artemisinin and arteanniun B in ArtH-Et_2_O were directly removed by the above method to prepare ArtH-Et_2_O without artemisinin (AHE-NA).

### 3.4. Animal Experiment

Three samples of different extracts were taken for parallel analysis. The contents of artemisinin in the different samples prepared above were determined using UPLC-PDA assay [19]. In the extract and ether-neutral-dry group, the absolute dosage of artemisinin was 10 mg/kg; in the ether-neutral-dry-without-artemisinin group, the low dose was 250 mg/kg, the medium dose was 500 mg/kg, and the high dose was 1000 mg/kg, respectively. The corresponding samples were weighed and dissolved by ultrasound with medicinal soybean oil (Aladdin).

Female ICR mice (20 ± 2 g) were purchased from the Vital Laboratory Animal Center (Beijing, China) and kept under SPF conditions at 25 °C with a 12 h light-dark cycle. Rodent strain *Plasmodium yoelii* (*P. yoelii*) obtained from the China Academy of Chinese Medical Sciences (Beijing, China) was maintained in ICR mice by syringe passage. All experiments were carried out in accordance with the guidelines approved by the Animal Ethics Committee of Capital Medical University.

The Peters’ four-day suppressive test [20,21] was used to test the antimalarial effect in mice. Blood smears were stained with 10% Giemsa-PBS staining solution for observation. The number of *P. yoelii* infections in at least 1000 erythrocytes of each mouse was counted, and the rates of *Plasmodium* infection and drug inhibition were calculated for different experimental groups. Statistical analysis of the data was performed using GraphPad Prism 8.0.2. One-way ANOVA and Tukey’s test for multiple comparisons between groups were used. Results are expressed as mean ± standard deviation (mean ± SD), *p* < 0.05 indicates statistical significance.

### 3.5. UPLC-Q-TOF-MS Measurement

Three copies of each sample were taken and dissolved into 1 mg/mL of acetonitrile and centrifuged at high speed for 10 min; all samples were filtered through a 0.22 µm syringe filter.

The sample determination was performed on a Waters Acquity UPLC system (Waters Corp., Milford, MA, USA) and the separation was based on a Waters Acquity UPLC column (2.1 mm × 100 mm, 1.7 μm, Waters). The autosampler was set at room temperature and the column temperature was maintained at 45 °C. A quantity of 0.1% formic acid-aqueous solution (A)—acetonitrile (B) was injected at a flow rate of 0.20 mL/min with a gradient program as follows: 35–60% B (0.0–3.0 min), 60% B (3.0–8.0 min), 60–100% B (8.0–10.0 min), 100% B (10.0–12.0 min), 100–65% B (12.0–12.1 min), 65% B (12.1–14.0 min).The injection volume was 2 μL.

Mass spectrometric detection for all samples was carried out on a Waters Xevo G2 Q-TOF mass spectrometer (Waters Corp., Milford, MA, USA) equipped with an ESI source, in positive- and negative-ion detection mode, mass analyzer for sensitivity analysis mode, and MS^E^ data acquisition mode (Low CE OFF, High CE 12–25 eV); mass range *m*/*z* 100–1200, scanning time 0.2 s, capillary voltage + 3.0 KV, sampling cone 10 V, cone gas flow 50 L/h, ion source compensation voltage 80 V. The ion source temperature desolvation gas temperature was located at 450 °C and the flow rate of the desolvation gas was 800 L/h. The real-time correction solution was 50% acetonitrile aqueous solution containing 0.1% formic acid and 200 pg/μL LE, spray voltage + 3.0 kV. The mass spectrometry data were collected using Masslynx 4.1 software (Waters Corp., Milford, MA, USA).

### 3.6. Data Analysis by UNIFI Platform

The UNIFI platform (Waters Corp., Milford, MA, USA) can combine data acquisition, peak extraction, molecular formula determination, database search and report generation to enable rapid and comprehensive qualitative analysis of chemical components. The natural compounds contained in *A. annua* were collected from offline and online databases (Pubchem, Chemicalbook, Chemspider) and the literature [22]. A total of 371 compounds, including sesquiterpenoids, flavonoids, monoterpenoids, polyterpenoids, and some other compounds, were collected and imported into the UNIFI database. MS data were imported into the UNIFI platform for processing and qualitative identification based on the retention and fragmentation pathways of the control.

### 3.7. Multivariate Statistical Analysis

Raw mass spectrometry data (raw) of different samples were imported into Progenesis QI (Waters Corp., Milford, MA, USA) software for peak alignment, peak extraction and peak area normalization to obtain a two-dimensional dataset of LC-MS data (One element of the dimensional information is the retention time and ion of the marker; the other is the peak area corresponding to the marker). The dataset was imported into *EZinfo* software (Waters Corp., Milford, MA, USA) for principal component analysis (PCA) and orthogonal partial least squares discriminant analysis (OPLS-DA). Each point in the PCA score plot represents an LC/MS analysis that can indicate trends in grouping between samples and the degree of dispersion within a sample group; OPLS-DA looks for differences between groups based on known groupings to obtain the S-Plot plot. Points closer to the top right and bottom left of the plot indicate greater contribution to differentiating between the two groups (points closer to the outside of the X-axis indicates that the marker is more different between the two groups; closer to 1 and −1 on the Y-axis indicates that the marker is more stable between the two groups, i.e., less different within the group). Markers with VIP > 5 and ANOVA *p* value < 0.05 in the target quadrant were screened.

## 4. Discussion

Based on the finding that artemisinin is not the only active antimalarial component in *A. annua* and the occurrence of signs of resistance to artemisinin, it would be interesting to determine what the non-artemisinin active components of *A. annua* are and which components of *A. annua* act synergistically with artemisinin.

Different *A. annua* samples were prepared and the content of artemisinin was determined. The content of artemisinin in different ether-neutral-dry samples (2% ArtL-Et_2_O, 2% ArtM-Et_2_O and 2% ArtH-Et_2_O) was lower than those in the corresponding ether extracts. This suggests that alkali treatment not only removed the acidic substances in the extract, but also destroyed the structure of artemisinin and transformed it into other substances. Liu’s study [8] supported this conclusion, finding that artemisinin underwent a quantitative structural transformation in alkaline solutions into an α, β-unsaturated ketonate.

The antimalarial activity of different extracts on mice was studied using Peters’ four-day suppressive test. In the extract and ether-neutral-dry group, the absolute dosage of artemisinin was 10 mg/kg, and in the ether-neutral-dry-without-artemisinin group, the low dose was 250 mg/kg, the medium dose was 500 mg/kg, and the high dose was 1000 mg/kg, respectively. The efficacy results enabled identification of differences in the antimalarial activity of the different samples. There was no significant difference in the antimalarial activity of ArtL-Et_2_O compared to the ART group, while ArtM-Et_2_O and ArtH-Et_2_O had enhanced antimalarial activity, suggesting that the differences in the natural composition of the substances in different *A. annua* samples led to a difference in efficacy.

Observing that ArtH-Et_2_O showed better antimalarial activity than ArtL-Et_2_O and ArtM-Et_2_O, we prepared AHE-NA and evaluated its efficacy. AHE-NA showed antimalarial activity, indicating the presence of natural non-artemisinin antimalarial active components in *A. annua*. This is one of the reasons for the efficiency of some extracts.

The antimalarial activity of ether-neutral-dry samples with reduced artemisinin content was higher than that of the ART group and the corresponding extracts. This suggests that the alkali treatment caused changes in the material composition of the extracts and did not enrich artemisinin through acidic substances removal. There were other positive effects of alkali treatment leading to differences in antimalarial activity. The ether-neutral drying-without-artemisinin sample efficacy results showed that non-artemisinin active ingredients are still present in the alkali-treated samples.

UPLC-Q-TOF-MS was used to obtain the mass spectrum data of all samples, and the UNIFI platform was used for identification to clarify what the non-artemisinin antimalarial active ingredients were and which substances were responsible for the differences in activity. A total of 24 flavonoids, 68 sesquiterpenoids and 21 other compounds were identified. A multivariate statistical method was used to screen the different markers with positive correlations. The information on the markers, combined with the results of the UNIFI, was used to determine the corresponding compounds with positive correlations with antimalarial activity. The differential compounds associated with antimalarial activity of ArtH-Et_2_O and ArtL-Et_2_O were artemisinin, arteanniun D, arteanniun I, 1-oxo-2β-[3-butanone]-3α-methyl-6β-[2-propanoic ac-id]-cyclohexane, 4α,5α-epoxy-6α-hydroxy amorphan-12-oic acid, 1-oxo-2β-[3-butanone]-3α-methyl-6β-[2-propanoic acid]-cyclohexane and mikanin. The differential compounds associated with antimalarial activity of 2% ArtH-ET_2_O and ArtH-Et_2_O were arteannuin I and arteannuin K. The differential compounds associated with antimalarial activity of 2% AHE-NA and 2% ALE-NA were 5-hydroxy-3,4′,6,7-tetramethoxyflavone, arteannuin L and artemetin. The differential compounds screened in this study were divided into two categories. One category was polymethoxyl flavonoids, such as 5-hydroxy-3,4′,6,7-tetramethoxyflavone, mikanin and artemetin; the other category was structural analogues of arteanniun B, such as arteannuin L and arteannuin K. The compound structures are shown in Figure 7. Zhang et al. and Li et al. found that arteanniun B in *A. annua* showed enhanced antimalarial effects for artemisinin [21,23]. It has also been reported that flavonoids could inhibit the metabolism of artemisinin resulting in enhanced antimalarial effects [10,24,25]. Screening of the shared compounds based on chromatograms revealed that 5α-hydroperoxy-eudesma-4(15),11-diene may be one of the antimalarial active compounds. In summary, this study has confirmed the existence of natural non-artemisinin antimalarial active ingredients in *A. annua*. The main reason for the synergistic effect of alkali treatment was not the enrichment of artemisinin, but the removal of impurities and the enrichment of synergistic substances. The antimalarial active ingredient groups of *A. annua* include two categories: one is artemisinin and non-artemisinin anti-malarial active components, among which the non-artemisinin antimalarial active components may include 5α-hydroperoxy-eudesma-4(15),11-diene. The other group is antimalarial synergistic ingredients, such as arteanniun B, arteanniun B analogues and polymethoxy flavonoids. The study identified a group of antimalarial active components of *A. annua*, which might lead to the development of new natural ACTs composed of artemisinin and other *A. annua* components. Further validation of the antimalarial activity of 5α-hydroperoxy-eudesma-4(15),11-diene and the effect of combination of antimalarial active ingredients with synergistic substances to produce antimalarial activity is necessary.

## 5. Conclusions

This study confirmed the existence of natural non-artemisinin antimalarial active ingredients in *A. annua*. The main reason for the synergistic effect of alkali treatment was not the enrichment of artemisinin, but the removal of impurities and the enrichment of synergistic substances. The antimalarial active components of *A. annua* include two categories: one is artemisinin and the other non-artemisinin antimalarial active components, among which the non-artemisinin antimalarial active components may include 5α-hydroperoxy-eudesma-4(15),11-diene. The other group is antimalarial synergistic ingredients, such as arteanniun B, arteanniun B analogues and polymethoxy flavonoids. This study provides the basis for establishing a natural combination of ACTs. 

## Figures and Tables

**Table 1:** Contents of artemisinin in different *Artemisia annua* L. samples. (*n* = 3).

Group	Mean Content/%	RSD/%
ArtL-Et_2_O	1.84	0.18
2% ArtL-Et_2_O	0.62	0.29
ArtM-Et_2_O	13.60	2.68
2% ArtM-Et_2_O	11.77	1.77
ArtH-Et_2_O	16.69	2.58
2% ArtH-Et_2_O	10.23	3.93

RSD: relative standard deviation, ArtL(M/H)-Et_2_O: ether extract of *Artemisia annua* L. with low/medium/high content of artemisinin, 2% ArtL(M/H)-Et_2_O: neutral dry of ArtL(M/H)-Et_2_O.

**Table 2:** LC-MS data of *Artemisia annua* L. based on UPLC-Q-TOF-MS/MS for identification of reference samples.

NO.	Component Name	Neutral Mass (Da)	Observed *m*/*z*	RT (min)	MS/MS
**1**	**Artenniun D**	282.1467	283.1655	2.70	265.1551, 247.1409, 219.1470
			281.1600 *		265.1625, 251.1826, 181.0640
**2**	**Chrysospleno D**	360.0845	361.0916	3.07	345.0744, 303.0629
			359.1078 *		344.0792, 329.0523
**3**	**Casticin**	374.1002	375.1073	3.90	360.1020, 345.0782, 317.0796
			373.1233 *		358.0995, 343.0695
**4**	**1-Oxo-2-[3-butanone]-3-methyl-6-[2-propanoic acid]-cyclohexane methyl ester**	268.1675	291.1691	4.26	219.1500, 191.1509, 173.1407, 145.1078
**5**	**Artenniun B**	248.1412	249.1589	4.47	231.1490, 185.1413
**6**	**Artemisinin**	282.1467	283.1655	5.10	265.1551, 247.1441, 237.1582, 229.1470, 219.1500
**7**	**Artenniun C**	266.1518	267.1716	5.24	249.1589, 231.1490, 207.1467
			265.1659 *		116.9371
**8**	**Artenniun I**	234.1620	235.1800	7.21	217.1672, 189.1727, 161.1393
			233.1733 *		116.9371
**9**	**Dihydroartemisinic acid**	236.1776	237.1960	8.53	219.1833, 201.1731, 163.1562
			235.1883 *		
**10**	**Artemisinic acid**	234.1620	235.1800	9.20	217.1672, 199.1551, 189.1727
			233.1733 *		

* indicates the value of the negative-ion mode.

**Table 3:** Identification of *Artemisia annua* L. compounds based on UPLC-Q-TOF-MS/MS. (*n* = 3).

NO.	Component Name	Neutral Mass (Da)	Observed *m*/*z*	Mass Error (mDa)	RT (min)	MS/MS
**Flavonoids**						
**1**	**Patuletin-3-*O*-glucoside**	494.1060	495.1092	−4.2	1.24	333.0584, 318.0336, 287.0535
**2**	**Astragalin**	448.1006	447.1125 *	0.6	1.27	285.1470, 257.1145
**3**	**Quercetin**	302.0427	303.0488	−1.1	2.18	287.0543
**4**	**4H-1-Benzopyran-4-one, 5,7,8-trihydroxy-2-(3-hydroxy-4-methoxyphenyl)-3-methoxy-**	346.0689	347.0757	−0.5	2.41	317.0650
**5**	**Patuletin**	332.0532	333.0591	−1.4	2.22	318.0346, 287.0545
		332.0532	331.0459 *	0.0	2.25	285.0410
**6**	**Kaempferol**	286.0477	287.0541	−0.9	2.67	153.0173, 229.0476
		286.0477	285.0400 *	−0.5	2.73	151.0031
**7**	**Chrysoeriol**	300.0634	301.0699	−0.8	2.70	286.0465, 258.0513, 257.0406
**8**	**Rhamnetin**	316.0583	317.0645	−1.1	2.77	302.042, 270.0513
		316.0583	315.0504 *	−0.7	2.82	299.01, 243.0306
**9**	**Axillarin**	346.0689	347.0753	−0.8	2.84	289.1403
		346.0689	345.0615 *	0.0	2.86	314.0454, 299.0194
**10**	**2,4′,5′-Trihydroxy-5′6,7-trimethoxyflavone**	360.0845	361.0916	−0.2	3.07	345.0604, 328.0575
**11**	**Chrysosplenol D**	360.0845	361.0916	−0.2	3.07	361.1007, 362.1008, 363.1022, 345.0730, 328.0613
**12**	**Eupatin**	360.0845	361.0913	−0.5	3.62	331.0854, 291.1586, 233.1570
**13**	**4H-1-Benzopyran-4-one, 2-(2,4-dihydroxyphenyl)-5-hydroxy-6,7-dimethoxy-**	330.0740	331.0805	−0.7	3.63	331.0854, 316.0571
**14**	**Penduletin**	344.0896	345.0963	−0.6	3.76	328.0634
		344.0896	343.0824 *	0.1	3.79	
**15**	**Casticin**	374.1002	375.1073	−0.2	3.90	377.1171, 376.1163, 375.1168
**16**	**Chrysosplenetin**	374.1002	397.0887	−0.7	3.91	397.0939, 375.1168, 317.0657
**17**	**Quercetagetin-6,7,3′,4′-tetramethylether**	374.1002	375.1076	0.1	4.00	
**18**	**Cirsimaritin**	314.0790	315.0846	−1.7	4.38	300.0607, 285.0376
**19**	**Mikanin**	344.0896	345.0967	−0.2	4.53	330.0771, 271.1332, 205.1633
		344.0896	343.0819 *	−0.4	4.57	328.0585, 313.0351, 285.0435
**20**	**Artemetin**	388.1158	389.1231	0.0	4.76	331.0806, 313.0702283.0598, 267.0644, 135.0436
**21**	**Rhamnocitrin**	300.0634	301.0705	−0.2	5.64	
**22**	**5-Hydroxy-3,4′,6,7-tetramethoxyflavone**	358.1053	359.1125	0.0	5.64	359.1201
**23**	**4H-1-Benzopyran-4-one, 3-hydroxy-6,7-dimethoxy-2-(4-methoxyphenyl)-**	328.0947	329.1020	0.0	6.92	314.0840
**Sesquiterpenoids**						
**1**	**Arteannuin M**	268.1675	291.1563	−0.4	2.27	235.1312, 217.1199, 207.1371
**2**	**Artemisinin B**	266.1518	265.1445 *	−0.1	2.41	251.1292, 241.1448, 235.1341
**3**	**Artenniun D**	282.1467	281.1389 *	−0.5	2.61	219.1348, 191.1049, 172.8616
**4**	**Artemisin**	262.1205	263.1262	−1.6	2.68	231.1366, 203.1422, 161.0951
**5**	***seco*** **-Cadinane**	266.1518	311.1493 *	−0.7	2.71	237.1488, 219.1002, 175.1120
**6**	**Dihydroxycadinanolide**	266.1518	265.1447 *	0.1	2.75	247.1341, 231.1396, 219.1021
**7**	**1-Oxo-2β-[3-butanone]-3α-methyl-6β-[2-propanoic acid]-cyclohexane**	254.1518	277.1409	−0.1	2.83	237.1582, 209.1525, 151.1111
		254.1518	253.1445 *	0.0	2.86	183.1025, 109.0670, 235.1340
**8**	**α-Hydroxysantonin**	262.1205	263.1213	−6.5	3.16	157.0635, 107.0488
**9**	**Arteannuin L**	250.1569	273.1456	−0.5	3.24	233.1528, 189.1261, 187.1474
**10**	**Dihydroartemisinic acid hydroperoxide**	268.1675	267.1600 *	−0.1	3.27	249.1496, 221.1547, 203.1440
**11**	**Arteannuin G**	282.1467	283.1504	−3.6	3.39	233.1131, 163.0741, 125.0936
**12**	**4α,5α-Epoxy-6α-hydroxy amorphan-12-oic acid**	268.1675	267.1597 *	−0.5	3.46	251.1648, 223.1343, 205.1593
**13**	**Norannuic acid formyl ester 15-nor-10-Hydroxy-oplopan-4-oic acid**	240.1725	285.1708 *	0.1	3.65	223.1343, 177.0919, 211.1345
**14**	**Arteannuin K**	250.1569	249.1487 *	−0.9	3.80	231.1391, 217.1231, 202.0994
**15**	**4-Amorphene-3,7-diol (3α,7α), acetate-**	280.2038	303.1906	−2.5	4.04	249.1478, 231.1362, 203.1417
**16**	**4-Amorphene-3,7-diol (3α,7α)**	238.1933	261.1807	−1.8	4.10	209.1517, 202.1641, 175.1106
**17**	**1-Oxo-2-[3-butanone]-3-methyl-6-[2-propanoic acid]-cyclohexane methyl ester**	268.1675	267.1599 *	−0.3	4.17	253.1449, 235.1339, 183.1024
**18**	**4-Amorphen,3,11-diol**	238.1933	261.1825	0.0	4.22	187.1473, 159.1164, 129.0690
**19**	**α-Epoxy-dihydroartemisinic**	252.1725	251.1351 *	−0.2	4.26	237.1491, 205.1596, 191.1440
**20**	**Arteannuin E**	250.1569	251.1634	−0.8	4.29	233.1510, 215.1402, 191.1041
**21**	**Artenniun B**	248.1412	271.1300	−0.4	4.47	205.1581
**22**	**3α,15-Dihydroxy cedrane**	238.1933	261.1831	0.6	4.60	209.1516, 175.1107, 147.1162
**23**	**Unknown**	232.1463	233.1531	−0.6	4.83	215.1416, 187.1472, 145.1004
**24**	**Arteannuin F**	250.1569	249.1488 *	−0.8	4.84	235.1340, 231.1385, 209.1179
**25**	**Abscisic acid**	264.1362	265.1433	−0.2	4.85	
		264.1362	263.1288 *	−0.1	4.85	
**26**	**5α-Hydroperoxy-eudesma-4(15),11-diene**	236.1776	237.1842	−0.7	5.05	221.1514, 219.1833, 201.1731
**27**	**Cubenol**	222.1984	261.1658	4.3	5.11	145.1007, 128.0618, 105.0697
**28**	**Artemisinin**	282.1467	281.1395 *	0.0	5.17	265.1451, 253.1448, 219.1389
**29**	**Arteanniun A**	206.1307	207.1375	−0.5	5.24	193.1578, 161.1315, 133.1007
**30**	**β-copaen-4α-ol**	234.1984	273.1659	4.3	5.25	203.1420, 185.1314, 159.1162
**31**	**3α-Hydroxy-4α,5α-epoxy-7-oxo-(8[7→6]-abeo-amorphane**	238.1569	239.1632	−1.0	5.26	203.1421, 179.1056, 133.1007
		238.1569	283.1549 *	−0.2	5.16	219.1389, 183.1025, 149.0969
**32**	**Artenniun C**	266.1518	265.1444 *	−0.1	5.30	247.134, 233.1175, 203.1435
**33**	**α-Epoxyartemisinic acid**	250.1569	249.1494 *	−0.3	5.35	205.1596
**34**	**artemisitene**	280.1311	281.1353	−3.1	5.44	247.1314, 209.1523
**35**	**Norannuic acid**	224.1412	247.1321	1.6	5.79	209.1525, 163.1107, 131.0848
**36**	**Arteannuin H**	250.1569	273.1436	−2.5	6.31	203.1415, 189.1258, 147.1158
**37**	**Nortaylorione**	220.1463	221.1526	−1.0	6.62	163.1105, 139.1106, 135.1156
**38**	**Deoxyarteannuin B**	232.1463	233.1531	−0.6	6.69	
**39**	**Annulide**	232.1463	233.1531	−0.6	7.00	215.1514, 187.1572
**40**	**Spathulenol**	220.1827	221.1887	−1.3	7.12	203.1789, 175.1476, 123.1156
**41**	**Artenniun I**	234.1620	233.1548 *	0.1	7.22	176.0819
**42**	**Dihydro-deoxyarteannuin B**	234.1620	235.1689	−0.4	7.26	189.1628, 161.1322
**43**	**Arteannuin J**	234.1620	235.1690	−0.3	7.61	217.1577, 189.1625, 161.1320
**44**	**Dihydroartemisinic acid**	236.1776	259.1662	−0.7	7.63	219.1736, 189.1625, 161.1320
**45**	**3-Isobutylcadin-4-en-11-ol**	308.2351	307.2275 *	−0.4	7.70	277.2167, 251.1658, 195.1385
**46**	**Nootkatone**	218.1671	219.1736	−0.7	8.51	203.1415, 163.1103, 123.0791
**47**	***trans*** **-α-bergamotyl acetic anhydride**	276.1725	277.1785	−1.3	8.81	233.1520, 205.1572, 235.1676
**48**	**Elemyl acetate**	264.2089	265.2144	−1.8	9.06	233.1522, 205.1572, 191.1410
**49**	**Artemisinic acid**	234.1620	235.1675	−1.8	9.19	175.1103
**50**	**Artemisinic aldehyde**	218.1671	219.1732	−1.1	9.34	202.1265
**51**	**Artemisinic acid methyl ester**	248.1776	293.1794 *	3.5	9.99	221.1545, 177.1279
**52**	**Occidentalolacetate**	246.1984	285.1657	4.2	10.04	189.1627, 163.1109
**53**	**α-Aromadendrene**	204.1878	205.1941	−1.0	10.15	
**54**	**6,7-dehydroartemisinic acid**	232.1463	233.1531	−0.5	10.25	205.1576, 187.1472, 175.1102
**55**	**14-Hydroxy-α-humulene**	262.1933	307.1920 *	0.5	10.28	221.1549, 191.1069, 151.1126
**56**	**4(15),5,11-Cadinatriene**	202.1722	203.1789	−0.5	10.32	177.1627, 175.1468, 173.1313
**57**	**14-Hydroxy-δ-cadinene**	220.1827	221.1890	−1.0	10.32	203.1788, 173.1316, 161.1316
**58**	**α-Longipinene**	204.1878	205.1963	1.2	10.33	191.1799, 175.1473, 161.1316
**59**	**β-Longipinene**	204.1878	205.1945	−0.6	10.57	187.1470, 158.1039
**60**	***cis*** **-Calamenene**	202.1722	203.1789	−0.5	10.71	173.0881, 145.1004, 115.0537
**61**	**α-Cubebene**	204.1878	205.1945	−0.6	10.99	159.11651, 145.10085, 131.0852
**62**	**Cedrylacetate**	264.2089	287.1962	−1.9	11.11	109.0644
**63**	**Cedra-8(15)-en-9α-ol acetate**	262.1933	285.1824	−0.1	11.30	231.1364, 219.1331, 173.1317
**64**	**Guaiazulene**	198.1409	199.1462	−1.9	11.40	185.1308, 143.0842, 128.0613
**65**	**α-Ylangene**	204.1878	227.1760	−1.1	11.41	161.1318, 147.1163, 129.0689
**66**	**Pregeijerene**	162.1409	185.1310	1.0	11.47	131.0846, 119.08495, 105.0694
**67**	**Cedra-8-en-13-ol, acetate**	248.1776	271.1640	−2.8	11.54	189.1250, 159.1163, 157.1003
**68**	**1β-Hydroxy-4(15),5-eudesmadiene**	220.1827	221.1901	0.1	11.73	207.1731, 180.1464, 146.0806
**Others**						
**1**	**3-Cyclohexene-1-methanol 2-hydroxy-α,α,4-trimethyl-, 1-acetate**	212.1412	235.1313	0.9	5.11	105.0697
**2**	***trans*** **-5-Hydroxy-2-isopropenyl-5-methylhex-3-en-1-ol**	170.1307	193.1204	0.5	5.26	157.1072, 121.1004, 107.0849
**3**	**2,6-Octadien-1-ol, 2,6-dimethyl-8-[(tetrahydro-2H-pyran-2-yl)oxy]-**	254.1882	253.1810 *	0.0	8.71	235.1700, 219.1872, 205.1598
**4**	**1,10-Oxy-α-myrcene hydroxide**	168.1150	191.1039	−0.3	10.25	119.0850, 105.0695
**5**	**2-Cyclohexen-1-ol, 3-methyl-6-(1-methylethylidene)-**	152.1201	175.1112	1.9	10.25	135.1221, 119.0850, 109.1001
**6**	**2,6-Dimethyl-1,3,5,7-octatetraene**	134.1096	135.1155	−1.3	10.27	107.0850, 105.0695
**7**	**2-Butenoic acid, 3-methyl-,(1S,2R,4S)-1,7,7-trimethylbicyclo [2.2.1] hept-2-yl ester**	236.1776	235.1709 *	0.5	10.30	221.1547, 179.1078, 137.0968
**8**	***p*** **-Menth-3-ene**	138.1409	161.1319	1.8	10.34	123.1158
**9**	**Menthol**	156.1514	195.1143	−0.3	10.75	143.0886, 135.1221, 109.1006
**10**	**Borneol isobutyrate**	224.1776	247.1683	1.5	11.11	139.0745, 123.0778, 123.0422
**11**	**Myrtenal**	150.1045	173.0941	0.4	11.16	121.1004, 105.0696
**12**	**Stigmasterol**	412.3705	413.3770	−0.8	11.32	217.1570, 199.1466, 147.1159, 128.0613
**13**	**2-Cyclohexen-1-one, 2-methyl-5-(1-methylcyclopropyl)-**	164.1201	187.1091	−0.2	11.33	
**14**	**Lavandulylacetate**	196.1463	219.1333	−2.2	11.37	122.0793
**15**	**Sabinene**	136.1252	159.1158	1.4	11.39	105.0694
**16**	**β-Amyrin acetate**	468.3967	491.3903	4.4	11.44	437.3354, 201.1628, 133.1008, 119.0854
**17**	**Bornylacetate**	196.1463	219.1344	−1.1	11.55	
**18**	**Carvone**	150.1045	173.0938	0.1	11.57	135.1198, 119.0909
**19**	**Fenchol**	154.1358	177.1250	0.0	11.62	137.1312, 122.0808
**20**	**(2*E*)-Hexadecene**	280.3130	303.3021	−0.1	11.86	163.1466, 135.1158, 121.1004, 107.0851
**21**	**Taraxasterone**	424.3705	425.3740	−3.8	12.84	409.3410, 201.1623, 161.1314, 133.1003

* indicates the value of the negative-ion mode.

**Table 4:** The differential chemical compositions positively correlated with antimalarial activity between ArtL-Et_2_O and ArtH-Et_2_O.

NO.	Primary ID	Neutral Mass (Da)	*m*/*z*	Rt (min)	Identification	VIP [1 + 1 + 0]
	**Positive ion mode**					
1	7.26_178.1355n	178.1355	161.1322	7.26	Arteanniun I	6.80
2	7.26_234.1612n	234.1612	235.1684	7.26	Arteanniun I	6.67
3	5.11_282.1456n	282.1456	265.1427	5.11	Artemisinin	6.09
4	2.85_276.1329n	254.15181	277.1402	2.85	1-Oxo-2β-[3-butanone]-3α-methyl-6β-[2-propanoic acid]-cyclohexane	5.64
5	5.10_246.1244n	246.1244	247.1322	5.10	Artemisinin	5.44
6	4.53_345.0959*m*/*z*		345.0959	4.53	Mikanin	5.34
7	5.10_236.1408n	236.1408	219.1375	5.10	Artemisinin	5.27
	Negative ion mode					
8	2.55_283.1527*m*/*z*		283.1527	2.55	Arteanniun D	7.54
9	3.44_267.1582*m*/*z*		267.1582	3.44	4α,5α-Epoxy-6α-hydroxy amorphan-12-oic acid	7.11
10	2.88_254.1498n	254.1498	253.1435	2.88	1-Oxo-2β-[3-butanone]-3α-methyl-6β-[2-propanoic acid]-cyclohexane	6.35

**Table 5:** The differential chemical compositions positively correlated with antimalarial activity between ArtH-Et_2_O and 2% ArtH-Et_2_O.

NO.	Primary ID	Neutral Mass (Da)	*m*/*z*	Rt/min	Identification	VIP [1 + 1 + 0]
1	7.26_234.1610n	234.1610	235.1682	7.26	Arteannuin I	6.76
2	7.26_178.1353n	178.1353	161.1321	7.26		6.67
3	3.86_250.1558n	250.1558	251.1631	3.86	Arteannuin K	5.35

**Table 6:** The differential chemical compositions positively correlated with antimalarial activity between 2% ALE-NA and 2% AHE-NA.

NO.	Primary ID	Neutral Mass (Da)	*m*/*z*	RT (min)	Identification	VIP [1 + 1 + 0]
1	5.64_358.1045n	358.1045	359.1117	5.64	5-Hydroxy-3,4′,6,7- tetramethoxyflavone	10.27
2	3.25_232.1452n		233.1525	3.25	Arteannuin L	5.50
3	3.25_187.1471 *m*/*z*		187.1471	3.25		5.71
4	4.75_388.1152n	388.1152	389.1225	4.75	Artemetin	5.34

## Data Availability

Data is available on request from the authors.

## Abbreviations

ACTs	Artemisinin-based combination therapies
ART	Artemisinin
ArtL(M/H)	*Artemisia annua* L. with low/medium/high content of artemisinin
ArtL(M/H)-Et_2_O	Ether extract of *Artemisia annua* L. with low/medium/high content of artemisinin
2% ArtL(M/H)-Et_2_O	Neutral dry of ArtL(M/H)-Et_2_O
2% AL(M/H)E-NA	Neutral dry of 2% ArtL(M/H)-Et_2_O without artemisinin
AHE-NA	ArtH-Et_2_O without artemisinin
PE	Petroleum ether
EA	Ethyl acetate
